# Extending integrate-and fire model neurons to account for the effects of weak electric fields in the presence of dendrites

**DOI:** 10.1186/1471-2202-16-S1-P185

**Published:** 2015-12-18

**Authors:** Florian Aspart, Josef Ladenbauer, Klaus Obermayer

**Affiliations:** 1 Neural Information Processing Group, Berlin Institute of Technology, Berlin, Germany; 2 Bernstein Center for Computational Neuroscience Berlin, Berlin, Germany

## 

Transcranial brain stimulation techniques have recently sparked a strong interest in understanding the effects of weak electric fields on neuronal network dynamics (e.g. [1,2]). The collective dynamics of large populations of coupled neurons can be efficiently studied using single-compartment (point) model neurons of the integrate-and-fire (IF) type [3], which allow for a systematic model reduction at the population level [4,5], as opposed to multi-compartment Hodgkin-Huxley type models and complex morphologies. However, existing point neuron models cannot adequately reproduce the effects of an electric field on the somatic membrane potential, which are influenced by the presence of dendritic processes [2].

Here, we present an extension for IF type point neuron models to take into account the subthreshold effects of an oscillating weak uniform extracellular field, similar to those generated in the brain by transcranial electrical stimulation [6]. Based on a "ball-and-stick" neuron model (i.e., a passive finite dendritic cable with a lumped soma at its end) we analytically calculate the somatic membrane polarization induced by a weak extracellular electric field using the cable equation. From this polarization we derive an equivalent input current for leaky IF as well as adaptive nonlinear IF point neurons, which explicitly depends on the (soma+dendrite) neuron model and electric field parameters. The extended point neuron model can well reproduce the relationships between electric field properties (intensity, frequency) and neuronal responses (membrane polarization, sensitivity and phase), as observed by simulations of neuron models with complex morphologies and reported in the experimental literature [7]. Our point neuron model extension is simple to implement and well suited for application in IF based neural networks.
